# Treatment decisions of patients with Class II Division 2 malocclusion and severe tooth wear: a systematic review

**DOI:** 10.1038/s41405-024-00248-x

**Published:** 2024-08-13

**Authors:** Yuhan Ma, Weijia Zhao, Sisi Zhang, Xiaoting Jin, Jianhao Xu, Baiping Fu, Ying Shi

**Affiliations:** Stomatology Hospital, School of Stomatology, Zhejiang University School of Medicine, Provincial Engineering Research Center for Oral Biomaterials and Devices, Zhejiang Provincial Clinical Research Center for Oral Diseases, Key Laboratory of Oral Biomedical Research of Zhejiang Province, Cancer Center of Zhejiang University, Hangzhou, Zhejiang China

**Keywords:** Oral diseases, Dentistry

## Abstract

**Background:**

The treatment strategy for patients with severe tooth wear associated with Class II Division 2 malocclusion remains a major challenge for dental practitioners.

**Objectives:**

To systematically review and summarize the literature on treatment strategies, restoration procedures and clinical outcomes for Class II Division 2 malocclusion patients with severe tooth wear.

**Methods:**

A literature review was conducted using Pubmed, Embase, the Cochrane Library, and Web of Science to identify eligible articles. Publications until October 16th, 2023 were searched independently and cross-checked by two researchers.

**Results:**

Of 1513 articles screened, 10 reports detailed treatment processes, including six males and four females aged 34–68 years old. Four articles recorded pre-treatment freeway space (FWS) values ranging from 5 to 9 mm. All ten cases had significant occlusal vertical dimension (OVD) loss and the increase in OVD after treatment ranged from 1 to 7 mm. Pre-prosthetic orthodontic treatment was performed in two cases, in one of which only the maxillary region was orthodontically treated. The most common restorations provided were full coverage restorations. In most cases, temporary restorations were applied before the permanent restorations for eight weeks to six months. Four different sequences of final restoration were proposed. Follow-up ranged from four months to six years and included seven patients, one of them showed symptoms of temporomandibular disorder (TMD).

**Conclusions:**

A multidisciplinary team (MDT) approach to treatment is recommended. Consideration of pre-prosthetic orthodontic treatment is essential. Commonly used cephalometric measurements for anterior teeth include the interincisal angle and collum angle. The increases in OVD ranging from 1 to 7 mm can be effectively accommodated. Temporary restorations are recommended to accommodate the OVD, and the transition periods of 8 weeks to 6 months help the patients adapted well. Four different sequences for final rehabilitation have demonstrated positive clinical outcomes. Full crown restorations have emerged as the preferred choice for the ultimate restoration of these patients.

## Introduction

Tooth wear is a common phenomenon that is defined as flattening of cusp tips or loss of incisal edges by physiological or pathological processes [1, 2]. Tooth wear results from mechanical factors like bruxism, chemical erosion from acidic foods, and biological influences such as saliva composition and flow rates [3]. The European expert consensus guidelines [4] define severe tooth wear as ‘tooth wear with substantial loss of tooth structure, with dentin exposure and significant loss (≥1/3) of the clinical crown’. Severe tooth wear may provoke some unfavorable effects including occlusal disharmony, decreased occlusal vertical dimension (OVD), pulpitis or hypersensitivity related to dentine exposure, poor esthetic, decrease in the masticatory function, and temporomandibular joint disorders [5].

Therapeutically, the choice of intervention depends on the severity and nature of the wear. In cases of mild wear, conservative treatments may involve dental bonding or resin composite restorations to restore lost tooth structure. For more extensive wear, especially involving occlusal surfaces, the use of partial or full coverage restorations, such as inlays, onlays, or crowns, may be considered to provide both functional and esthetic rehabilitation [6]. One significant factor that exacerbates tooth wear and complicates its management is malocclusion, particularly Class II Division 2 (Class II/2) malocclusion. This type of malocclusion, characterized by the lingual inclination of the maxillary incisors and deep overbites [7], often exhibits severe tooth wear [8]. Abnormal tooth friction and bite relationships, coupled with tooth tilting and asymmetry, contribute to uneven wear patterns. The instability of occlusion complicates the restoration process, necessitating solutions for bite balance and stability [9]. The increased complexity requires interdisciplinary collaboration among dental specialists, including orthodontists, prosthodontists, periodontists and so on. Orthodontists play a crucial role in realigning teeth to achieve proper occlusion, while prosthodontists focus on restoring tooth structure and esthetics. This collaborative approach is necessary to ensure a more stable and effective outcome.

The treatment strategy for patients with severe tooth wear associated with Class II/2 malocclusion is still up for debate. This study will systematically review and summarize literature on treatment strategies, restoration procedures and clinical outcomes for Class II/2 malocclusion patients with severe tooth wear.

## Material and methods

The systematic search was performed according to PRISMA guidelines [10]. It was conducted on the following electronic databases until 16th of October 2023: Medline (PubMed), Embase, the Cochrane Library, and Web of Science Core Collection. The database Medline (PubMed) was searched with the strategy in Table 1. For the other three databases, comparable terms were used but modified to be suitable for the specific criteria of the particular database.

**Table 1:** Searching strategy for PubMed.

Search	Query
**#1**	**“Malocclusion, Angle Class II”[Mesh]** OR (Class II Division 2[Title/abstract]) OR (Class II, Division 2[Title/abstract]) OR (Class II div. 2[Title/abstract]) OR (Class II div 2[Title/abstract]) OR (Angle Class II, Division 2[Title/abstract]) OR (Angle’s Class II, Division 2[Title/abstract]) OR (Class II Malocclusion, Division 2[Title/abstract]) OR (Class II/2 malocclusion[Title/abstract]) OR (II/2 malocclusion[Title/abstract]) OR (II/2 cover-bite[Title/abstract]) OR (II/2 deep-overbite[Title/abstract]) OR (Class II deep bite[Title/abstract])
**#2**	**“Overbite”[Mesh]** OR (overbites[Title/abstract]) OR (over bite[Title/abstract]) OR (over-bites[Title/abstract]) OR (over-bite[Title/abstract]) OR (deep-bite[Title/abstract]) OR (deep-bites[Title/abstract]) OR (deep bite[Title/abstract]) OR (deep bites[Title/abstract]) OR (cover-bite[Title/abstract]) OR (over-jet[Title/abstract]) OR (overjet[Title/abstract]) OR (overjets[Title/abstract]) OR (overlap[Title/abstract])
**#3**	**“Tooth Abrasion”[Mesh] OR “Tooth Attrition”[Mesh]** OR (abrasion[Title/abstract]) OR (abrasions[Title/abstract]) OR (attrition[Title/abstract]) OR (attritions[Title/abstract]) OR (wears[Title/abstract]) OR (worn[Title/abstract]) OR (wear[Title/abstract]) OR (erosion[Title/abstract])
**#4**	**#2 AND #3**
**#5**	**#1 OR #4**
**#6**	**“Mouth Rehabilitation”[Mesh] OR “Dental Articulators” [Mesh] OR “Vertical Dimension” [Mesh]** OR (Mouth rehabilitations[Title/abstract]) OR (Full-Mouth rehabilitation[Title/abstract]) OR (Full mouth rehabilitation[Title/abstract]) OR (occlusal rehabilitation[Title/abstract]) OR (occlusal reconstruction[Title/abstract]) OR (rehabilitation[Title/abstract]) OR (reconstruction[Title/abstract]) OR (mouth reconstruction[Title/abstract]) OR (fixed reconstruction[Title/abstract]) OR (restore occlusion[Title/abstract]) OR (restoration[Title/abstract]) OR (articulator[Title/abstract]) OR (articulators[Title/abstract]) OR (virtual articulators[Title/abstract]) OR (anterior guidance[Title/abstract]) OR (Mandibular rest position[Title/abstract])
**#7**	**#5 AND #6**

All databases were searched independently and cross-checked by two researchers (Y.M. and W.Z.). Any disagreements regarding the suitability of individual articles were resolved by discussion. The reference lists in the articles included in the study were further screened for additional eligible publications. There were no limitations on the types of articles. All articles reporting on one or multiple cases of Class II/2 patients with severe tooth wear were included. Articles were excluded based on the title, abstract, or full text. The detailed inclusion and exclusion criteria are outlined in Table 2.

**Table 2:** Inclusion and Exclusion criteria.

Criterion	Inclusion	Exclusion
Population	Patients with Class II Division 2 malocclusion and severe tooth wear	Patients with malocclusion other than Class II Division 2, absence of severe tooth wear
Setting	Any	N/A
Study designs	All study designs	N/A
Publication type	Peer review of original research (including reviews and case reports)	Opinion pieces, editorials, magazine articles
Outcomes	Articles that present treatment methods of Class II Division 2 patients with severe tooth wear through clinical cases	Articles that did not address clinical cases of patients with Class II Division 2 malocclusion and sever tooth wear
Language	Articles written in all languages	N/A
Availability	Full text available	Not full text available
Date	All articles from January 1990 to October 2023	Anything outside of this range

The risk of bias and quality of the included studies were independently assessed by two researchers (Y.M. and W.Z.) using the Joanna Briggs Institute (JBI) Critical Appraisal Checklist for case reports [11]. Any discrepancies were addressed by discussion or by consultation with a third researcher (Y.S.). The JBI checklist for case reports appraises studies based on an eight‐item scale, which includes the participants’ demographic characteristics, medical history, present clinical state, description of the diagnostic tests, treatment, post‐intervention clinical status, adverse occurrences, and the provision of takeaway lessons.

Two researchers independently performed data extraction from the eligible articles. Each paper was critically reviewed, and the following data were extracted: age and gender of the patient at presentation, freeway space (FWS) before treatment, the increase in OVD after treatment, description of orthodontic treatment, protocol for the prosthetic treatment and eventual follow-up duration.

## Results

**Fig. 1:**
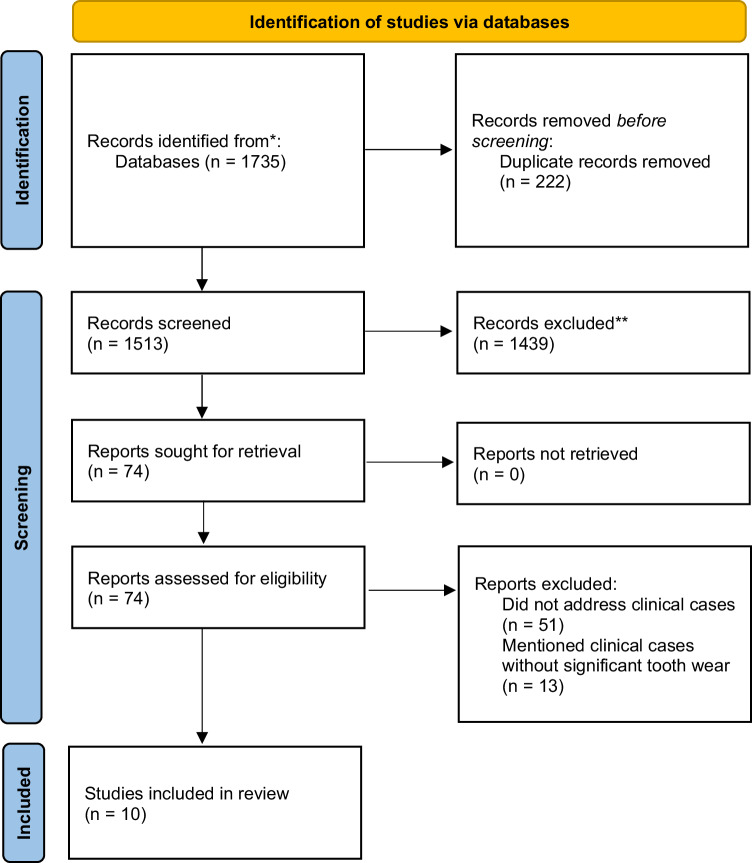
PRISMA flow diagram illustrating literature search and selection process.

Seventy-four publications were found in relation to Class II/2 malocclusion with severe tooth wear. However, fifty-one publications did not address clinical cases. Thirteen publications contained clinical cases in which the tooth wear did not meet the criteria for significance. The remaining ten articles that provided a detailed description of full mouth rehabilitation of Class II/2 patients with significant tooth wear were included. The flowchart of the literature search is presented in Fig. 1.

Detailed information on the clinical cases is presented in Tables 3–5. Ten clinical cases, including six males and four females aged 34–68 years old were included. Eight of the cases were from the last decade. The chief complaints of the patients varied and were dominated by tooth wear and pain. Most patients had no TMJ symptoms at the initial examination. All patients had inwardly inclined anterior teeth as well as deep overbite, and the majority of the patients had a malocclusion in the midline and intraoral restorations. Posterior teeth were present but incomplete in half of the patients and partially missing in the other half. Dental wear was more prevalent on the anterior teeth, especially on the palatal surfaces of the maxillary anterior teeth and the labial surfaces of the mandibular anterior teeth. The most common restorations provided were full coverage restorations (nine patients). Four patients had veneers or onlays as the restorative options [12–15] and implant restorations were also adopted in two patients [1, 15]. In most cases, temporary restorations were applied before the final restoration to ensure the patient could adapt to the new vertical height (eight patients) [12, 15–21]. The adaptation period for temporary restorations varies from eight weeks to six months. Occlusal splints were made in four cases to prevent parafunction and recurrence [12, 14, 19, 20]. Regarding the final restoration sequence, four cases restored anterior and posterior teeth simultaneously [13, 16, 17, 21]. Four cases restored anterior teeth first and then posterior teeth [12, 14, 15, 18] and one case used the opposite approach [19], restoring posterior teeth first and then anterior teeth. In another case [20], a new restorative approach was proposed, in which the left posterior teeth were restored first, followed by the anterior tooth, and finally the right posterior teeth were completed. Four articles recorded pre-treatment FWS values ranging from 5 to 9 mm [12, 18–20]. All ten cases had significant OVD loss and the increase in OVD after treatment ranged from 1–7 mm. Pre-prosthetic orthodontic treatment was performed in two cases [14, 15], in one of which only the maxillary region was orthodontically treated [15]. Follow-up ranged from four months to six years and included seven of the patients. One patient reported a clicking sound in opening at a one-month review, a dual stabilized appliance was fabricated and no signs of TMD were noted after three years of follow-up [12].

**Table 3:** Clinical presentations of the patients at initial examination.

Author	Age	Gender	Chief complaint	TMD	Intraoral restorations	Posterior teeth exist	Main wear zones
Constantinescu et al. [17]	58	F	Severe headache resistant to all common painkillers	Y	Y	N	upper and lower incisors
Hasanzade [12]	34	M	Moderate pain in posterior gingival tissue of upper teeth related to tapping force of lower anterior teeth, and esthetic and functional problems	N	Y	Y	labial surface of lower incisors
Tunkiwala [15]	62	M	Wear of his lower anterior teeth	N	Y	Y	labial surfaces of the mandibular anterior teeth and palatal surfaces of the maxillary anterior teeth
Seo [18]	68	M	Loss of maxillary posterior teeth	N	Y	N	lower incisors
Zhao [20]	50	F	Chewing pain in the left posterior teeth	N	Y	N	anterior and posterior teeth
Bosch [13]	47	M	Increasing pain and hypersensitivity in multiple teeth, and general dissatisfaction with the esthetics of his teeth	N	N	Y	labial surface of lower incisors
Ergun [19]	40	M	Inability to chew, problems with facial appearance, and replacement of missing teeth	N	N	N	upper and lower incisors
Dinning [14]	54	F	Improve her bite and tooth alignment	N	Y	Y	upper and lower incisors
Balshi [16]	67	M	Loose teeth and facial collapse	N	Y	N	lower incisors
Capp [21]	53	F	Severe loss of tooth structure	N	Y	Y	palatal surfaces of the maxillary central and lateral incisors and right canine

*M* male, *F* female, *Y* yes, *N* no.

**Table 4:** Types of restorations and the sequences of occlusal rehabilitation.

Restorative Treatment							
Author	Temporary restoration	Onlay/ Venner	Implant restoration	Full coverage restoration	Removable partial denture	Splint	Sequence
Constantinescu [17]	>6 m	N	N	Y	N	N	simultaneous
Hasanzade [12]	5 m	Y	N	Y	N	Y	LA-UA-P
Tunkiwala [15]	8w	Y	Y	Y	N	N	UA-LA-P
Seo [18]	10w	N	N	Y	Y	N	A-P
Zhao [20]	3 m	N	N	Y	N	Y	Left P-A-Right P
Bosch [13]	NR	Y	N	N	N	N	simultaneous
Ergun [19]	3 m	N	N	Y	N	Y	P-A
Dinning [14]	NR	Y	N	Y	N	Y	A-UP-LP
Balshi [16]	3 m	N	Y	Y	N	N	simultaneous
Capp [21]	2 m	N	N	Y	N	N	simultaneous

*A* anterior teeth, *P* posterior teeth, *U* maxillary teeth, *L* mandibular teeth. *N* No, *Y* Yes, *NR* not reported.

**Table 5:** Specific parameters, disciplines, and clinical outcomes of the restorative procedure.

Author	FWS before treatment (mm)	The increase in OVD (mm)	Follow Up	Clinical Outcomes	MDT
Constantinescu et al. [17]	NR	7	6 m	No complications	Endodontics, Prosthodontics
Hasanzade [12]	5	1-2	3 y	Patient reported a clicking sound in opening at a one-month review, a dual stabilized appliance was fabricated, and no complications were noted after 3-year follow-up	Prosthodontics
Tunkiwala [15]	NR	5.5	6 y	No complications	Endodontics, Orthodontics, Implantology, Prosthodontics
Seo [18]	7-8	4	6 m	No complications	Prosthodontics
Zhao [20]	8-9	5-6	4 m	No complications	Endodontics, Apical surgery, Prosthodontics
Bosch [13]	NR	3	NR	No complications	Endodontics, Prosthodontics
Ergun [19]	7	4-5	2 y	No complications	Prosthodontics
Dinning [14]	NR	1	NR	No complications	Endodontics, Orthodontics, Prosthodontics
Balshi [16]	NR	5	5 y	No complications	Implantology, Prosthodontics
Capp [21]	NR	3-4	NR	No complications	Prosthodontics
TOTAL	5–9 mm	1–7 mm	4m–6y		

*NR* not reported, *m* month, *y* year.

## Discussion

### Clinical Commonalities

The characteristics of Class II/2 patients are inwardly inclined incisors and deep overbite [22]. These patients have a special open and closed jaw movement pattern that corresponds to a special wear pattern. In these patients, the upper anterior teeth restrict the anterior and lateral movements of the lower jaw, creating a posteriorly rotated open and closed mouth movement pattern, resulting in wear of the lingual surface of the upper anterior teeth and the labial surface of the lower anterior teeth, and crater-like defects on the occlusal surfaces of the posterior teeth. These patients often suffer from severe tooth wear and restoration breakdown due to the characteristic jaw movement and high occlusal forces. In the reviewed literature, the most prevalent site of wear was observed on the lower incisors, followed by the upper incisors and posterior teeth. Deep bite is another feature of Class II/2 patients [23]. This type of incisal relationship can be problematic to both the patient and the dentist if it results in soft tissue trauma or tooth wear. At the same time, dentoalveolar compensation happens continuously and is accelerated by wear problems, eliminating the space required for reconstruction of worn teeth [24].

The pattern of hypodontia may further complicate the treatment of Class II/2 patients. It was found that the prevalence of permanent hypodontia was twice as high in Class II/2 patients as in general orthodontic patients, with a higher prevalence of mandibular second premolar, maxillary lateral incisor, and third molar [25]. Developmental dental anomalies, including tooth agenesis, have been suggested to be an anatomic characteristic of patients with Class II/2 malocclusion.

### Pre-prosthetic considerations

#### MDT

The condition of patients with Class II/2 malocclusion and severe tooth wear is intricate, often necessitating multidisciplinary combined treatment (MDT), involving specialties such as endodontics, periodontics, orthodontics, prosthodontics and maxillofacial surgery. Hence, an imperative exists to establish an effective MDT protocol from the outset, aiming to optimize treatment duration and enhance the overall healthcare experience for patients. For example, in the case study by Tunkiwala [15], the patient underwent initial maxillary orthodontic treatment followed by dental impressions. During the wax-up period, root canal treatment of some teeth and the first-stage implant operation for missing teeth were completed, and the temporary restorations were made three weeks later.

Various factors, including the patient’s needs, financial resources, level of motivation, and availability of time, play a significant role in determining appropriate treatment options. Additionally, the patient’s age should be taken into account when formulating a treatment plan. Clinical decisions are influenced by considerations such as life expectancy, treatment duration, patient comfort, adherence to treatment recommendations, and esthetic outcomes. Elderly patients frequently express a preference for fixed or removable restorations over more extensive and complex treatment modalities like orthodontic procedures and dental implant placement. Of the ten patients included, only two received pre-restorative orthodontic treatment [14, 15], two received implantations [15, 16], while the majority of the patients opted for full crown or veneer restorations, and one had a partial removable denture [18].

#### Orthodontic treatment

Restorative modifications alter tooth morphology but fall short in correcting malocclusion in Class II/2 patients or addressing underlying factors of significant tooth wear. Thus, orthodontic treatment should be given due consideration. Aligning the teeth through orthodontic treatment has several advantages: (1) it opens the occlusion and facilitates the seating of the restoration. (2) It aids in the alignment of the occlusal plane and the transfer of occlusal forces through the long axis of the teeth [26]. (3) it alters the position of the condylar process, facilitates the release of abnormal pressure on the posterior portion of the TMJ, and improves joint function [27].

In the management of Class II/2 malocclusion, several cephalometric assessment parameters play a crucial role. Among these, the maintenance of a proper interincisal angle is recognized as a critical factor in preventing relapse of deep bite, which refers to the angle formed between the upper and lower central incisor edges when the jaws are closed. Berg et al [28] found that an interincisal angle of less than 140 degrees was crucial for long-term stability. Collum angle (CA) is regarded as another significant factor for evaluating Class II/2 anterior teeth, which refers to the complementary angle of the crown-root angle [29]. The Class II/2 patients have the largest CA, with a mean value of 10.6° [30].

Two of the ten cases underwent pre-prosthetic orthodontic treatment, one of which involved only the upper jaw, which lasted 6 months [15]. The goal was to correct the inclination of the upper anterior teeth and improve the overbite to eliminate anterior friction. This also would make the treatment conservative because the overall preparation of teeth would be lessened and within the existing enamel after completion of orthodontic therapy. The cephalometric parameters were drastically improved after the treatment. The maxillary incisors were anteriorly inclined and the roots were palatally displaced by 3 mm. The lower anterior facial height increased by 5.5 mm, with posterior displacement of the chin by 3 mm. Maxillary and mandibular incisors demonstrated a normal relationship.

Orthognathic surgical treatment also needs to be considered when there are significant skeletal differences and orthodontic treatment cannot achieve good results. The maxillary anterior teeth of Class II/2 patients have relatively long crowns, short roots, reduced labio-palatal thickness, and altered crown-root angles. These dental features may impose severe limitations on the amount of torque that may be applied to the teeth, the speed of movement, and limit orthodontic outcomes [31]. Furthermore, if the patient has already experienced gingival recession, using orthodontics alone may worsen the periodontal condition, so a combined orthognathic approach may be necessary in such situation [2].

#### OVD

Severe tooth wear results in inadequate space for restorative interventions, necessitating an increase in vertical dimension. Dahl et al. proposed a successful range of increased vertical dimension of 1.8–4.7 mm. Many studies have since shown that patients adapt well when the increase in OVD is within 5 mm [22]. In this review, the increase in OVD ranged from 1 to 7 mm, and all of patients adapted well to the new height. In cases of Class II/2 patients, there is an argument advocating for minimal increases in vertical dimension, as long as both esthetics and function are maintained.

There are many different ways for the dentist to determine the increased value in OVD, the most commonly used is the FWS method, which was promoted by Niswonger [32]. The distance gap between occlusal vertical dimension (OVD) and rest vertical dimension (RVD) is called freeway space (FWS) [33]. However, aging can induce a loss in muscle tone, which can affect RVD [34]. So the value of FWS is a range that will vary from patient to patient, with an average value of around 3 mm. Silverman et al [35] discovered another way of determining OVD, which is around 1 mm when the patient pronounces the letter S.

Dahl devised the most prevalent approach to increase OVD, which uses Co - Cr appliances that lay on the palatal surface of the maxillary arch. The idea behind this device is to intrude anterior teeth and extrude posterior teeth in order to create space for restoration and improve interocclusal space [36]. Some other dentists improve the vertical dimension by restoring the occlusal surface of posterior teeth, freeing up room for anterior tooth restoration. Francesca et al [37] demonstrated a three-step procedure for restoring vertical dimension. The first step is to build an anterior mock-up for esthetic purposes and occlusion plane assessment, followed by the second step of making a posterior mock-up for posterior support. In the third and last phase, anterior guidance was created.

### Temporary restoration

Temporary restorations were predominantly utilized, facilitating dentists to make minor adjustments and providing patients with the opportunity to seek input from their social circle regarding the anticipated esthetic outcome [38]. Additionally, this interim measure allows patients to adjust to the new vertical dimension, potentially averting irreversible complications during the final restoration’s cementation process.

In the included literature, cases with a vertical dimension exceeding 3 mm all underwent temporary restoration [15–21]. Among cases with a vertical dimension less than 3 mm, one study [12] employed temporary restoration while two studies did not [13, 14]. This suggests that temporary restoration is recommended when the vertical dimension exceeds 3 mm, whereas, for dimensions less than 3 mm, its use may be discretionary, contingent upon individual patient considerations. The individualized consideration of patients’ unique circumstances and varying levels of prosthesis adaptation is imperative [39]. Among the ten cases examined, the minimum duration for temporary restoration was 8 weeks.

### Permanent restoration

Full crown restorations were predominantly utilized in the permanent restoration of patients with Class II/2 malocclusion, likely due to their superior mechanical properties that aid in managing abnormal occlusal forces. Despite the lack of consensus on the optimal sequence for permanent restorations, the outcomes of various procedures in 10 clinical cases were successful. Four cases restored anterior and posterior teeth simultaneously [13, 15, 16, 21]. Four cases restored anterior teeth first and then posterior teeth [12, 14, 15, 18], and one case used the opposite approach [19], restoring posterior teeth first and then anterior teeth. Zhao et al [20] proposed another restorative approach through a clinical case, in which the left posterior teeth were restored first, followed by anterior guidance, and finally the right posterior teeth were completed.

### Treatment stability

The changes in OVD are usually well tolerated by patients, but sometimes complications can occur. These include TMD, muscle problems, broken teeth, voice disorders, and excessive stress that leads to receding gums [29]. In one of the cases [12], the patient reported a clicking sound in opening at a review appointment one month after the restoration, and a dual stabilization appliance was fabricated for him. After a 3-year follow-up, no evidence of bone loss or loss of OVD, and no signs of TMD were noted.

Recurrence of malocclusion is common in Class II/2 patients after orthodontic treatment. The most common recurrences are deep overbite and upper incisor inclination [40]. Recurrence may be associated with persistent habituation or increased muscle activity. Modification of muscle activity by myotomy may be expected to prevent recurrence in these patients. T. Mücke et al [41] found that botulinum toxin injections are effective in preventing recurrence in adult patients with Class II skeletal patterns and that may be an alternative to traditional myotomy.

Prevention is better than cure. Early intervention should be considered depending on the patient’s functional and esthetic goals [42], especially in trauma-related Class II/2 malocclusions. To address and mitigate the potential risks, it is imperative to identify young patients at high risk of trauma and initiate suitable orthodontic interventions during their early ages.

### Treatment decisions

**Fig. 2:**
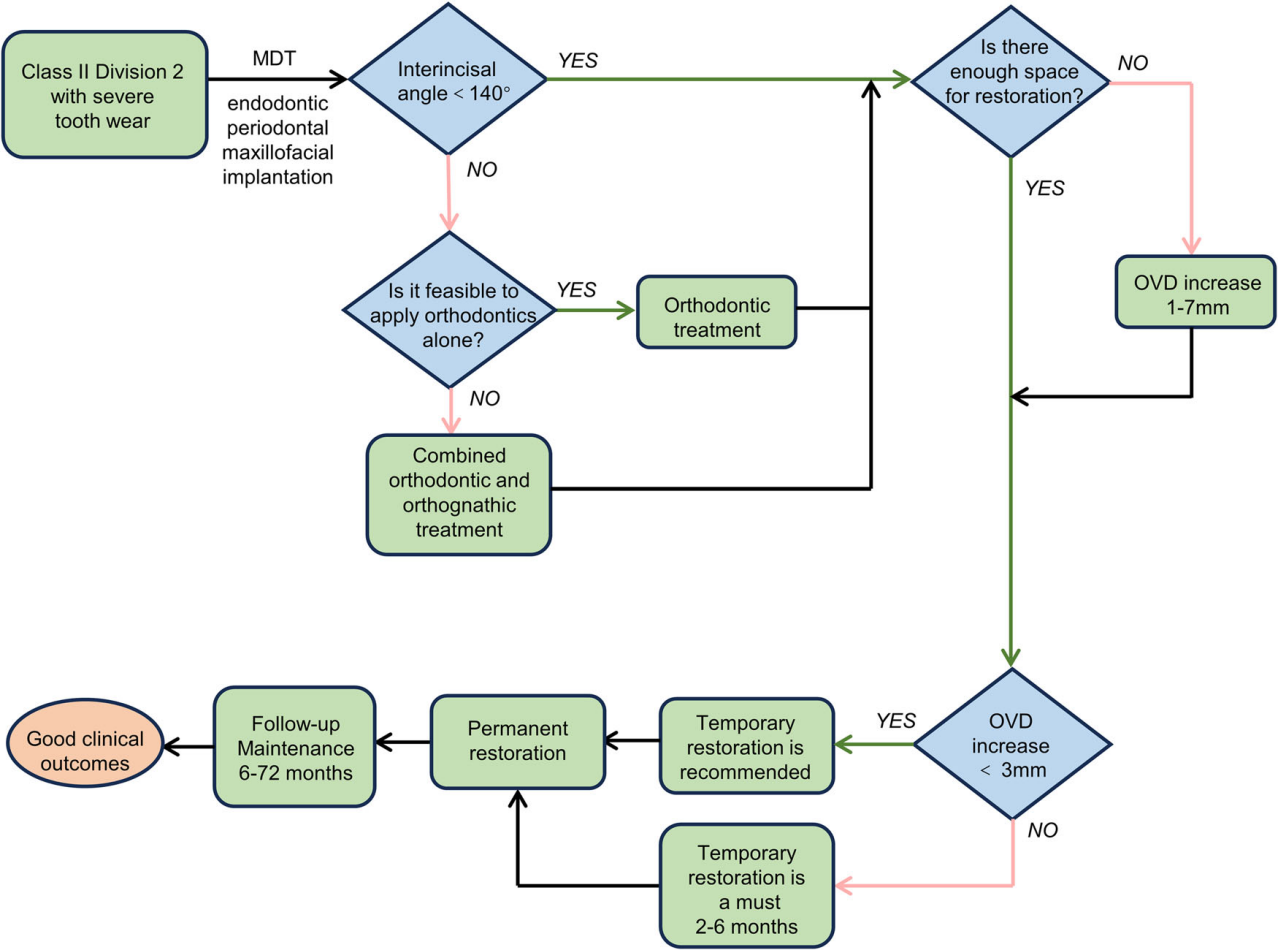
Treatment decision tree for Class II Division 2 patients with severe tooth wear based on the included articles.

Based on the above discussion, the treatment decision tree for patient with severe tooth wear and Class II/2 malocclusion is summarized in Fig. 2. Patients with severe tooth wear and Class II/2 malocclusion should undergo comprehensive evaluation and interdisciplinary treatment planning before initiation of therapy. Currently, there is no consensus on when orthodontic treatment should be included. This study suggests that considering the long-term stability of restoration, orthodontic treatment should be included when the interincisal angle exceeds 140°. When there is insufficient space for restoration, it is necessary to increase OVD. Included studies suggest increasing OVD by 1–7 mm. For OVD increases greater than 3 mm, temporary restorations over 2–6 months are necessary for patient adaptation. When OVD increases are less than 3 mm, the need for temporary restorations can be assessed based on the situation, but is usually recommended. There are various sequences for permanent restoration, yet consensus has not been reached. Regular follow-up is required after treatment is completed.

### Deficiency and prospect

Due to the restricted number of original studies, only ten case reports could be included, so the results should be interpreted with caution. Future research could incorporate larger sample sizes and longitudinal studies to enhance the robustness of evidence and deepen insights into treatment decisions for Class II/2 patients.

## Conclusion

Limited clinical cases of Class II/2 patients with severe tooth wear have been documented thus far. Findings from these ten cases indicate that a multidisciplinary team approach to treatment is recommended, providing efficiency advantages. Consideration of pre-prosthetic orthodontic treatment is essential. Commonly used cephalometric measurements for anterior teeth include the interincisal angle and collum angle. The increases in OVD ranging from 1 to 7 mm can be effectively accommodated. Temporary restorations are recommended to accommodate the OVD, and the transition periods of 8 weeks to 6 months help the patients adapt well. Four different sequences for final rehabilitation have demonstrated positive clinical outcomes. Full crown restorations have emerged as the preferred choice for the ultimate restoration of these patients.

## Data Availability

All data generated or analyzed during this study are included in the manuscript.
